# Effectiveness of a self-help guide during a temporary alcohol abstinence challenge: a randomized controlled trial

**DOI:** 10.1093/alcalc/agae034

**Published:** 2024-05-26

**Authors:** Annelien Esselink, Andrea D Rozema, Nathalie Kools, Tim Van Den Berk, Rob H L M Bovens, Jolanda J P Mathijssen

**Affiliations:** Tranzo Scientific Centre for Care and Wellbeing, Tilburg School of Social and Behavioural Sciences, Tilburg University, PO box 90153, 5000 LE Tilburg, The Netherlands; Tranzo Scientific Centre for Care and Wellbeing, Tilburg School of Social and Behavioural Sciences, Tilburg University, PO box 90153, 5000 LE Tilburg, The Netherlands; Tranzo Scientific Centre for Care and Wellbeing, Tilburg School of Social and Behavioural Sciences, Tilburg University, PO box 90153, 5000 LE Tilburg, The Netherlands; Gewoontegedrag, Toernooiveld 300, 6525 EC Nijmegen, The Netherlands; Tranzo Scientific Centre for Care and Wellbeing, Tilburg School of Social and Behavioural Sciences, Tilburg University, PO box 90153, 5000 LE Tilburg, The Netherlands; Positive Lifestyle Foundation, Ringbaan-Oost 298, 5018AL Tilburg, The Netherlands; Tranzo Scientific Centre for Care and Wellbeing, Tilburg School of Social and Behavioural Sciences, Tilburg University, PO box 90153, 5000 LE Tilburg, The Netherlands

**Keywords:** randomized controlled trial, alcohol drinking, temporary abstinence challenge, internet-based intervention, behaviour change

## Abstract

**Background:**

The popularity of temporary abstinence challenges (TACs) concerning alcohol consumption is increasing. Support is found to be essential for participants to help them get through a challenge. This study aimed to evaluate the additional effect of a self-help guide, based on health behaviour theories and behaviour change techniques, on (i) successful completion of a TAC and (ii) changes in drinking refusal self-efficacy (DRSE), behavioural automaticity, craving, and alcohol consumption.

**Methods:**

A randomized controlled trial was performed (OSF registries: OSF.IO/B95VU). NoThanks participants received a questionnaire before the TAC (T0) and 8 months after the TAC (T1). Out of a subgroup of 1308 respondents who were interested in additional support, 652 were randomly assigned to receive the guide (experimental group), and 656 did not receive any additional support (control group). Logistic regressions and (generalized) linear mixed model analyses were used.

**Results:**

After 8 months, all participants showed a significant decrease in behavioural automaticity, craving, and alcohol consumption, irrespective of group assignment. No significant changes were observed in the DRSE. This degree of change over time in behavioural automaticity, craving, and alcohol consumption did not differ between the experimental and control group. Sensitivity analyses with participants in the experimental group, who differed in exposure to the guide, did not show differences either.

**Conclusion:**

The self-help guide, and how it was designed, added no value to the TAC. Future research should focus on more bottom-up, customized support and explore what (different subgroups of) participants think they need as extra support during a TAC.

## Teaser

**Short Summary:** An online self-help guide was developed for motivated participants of a temporary abstinence challenge aimed at longer-term behavioural change. An RCT was performed, which showed that the self-help guide had no additional effect on the successful completion of the challenge, long-term self-efficacy, behavioural automaticity, craving, or alcohol consumption.

## Introduction

In many European countries, the use of alcohol is deeply embedded (Kummetat et al. 2022). For example, 73.8% of the European population above the age of 15 reported drinking in the past year, of whom 8.4% reported drinking every day in 2019 (Eurostat 2022). Although alcohol consumption is generally socially accepted in European countries, the risks of (excessive) alcohol consumption are often unknown or underestimated by people drinking alcohol (Schouten et al. 2021).

Many countries are increasingly running campaigns challenging people to stop drinking alcohol for 30 or more days, such as Dry January in the UK, Tournee Minérale in Belgium, and NoThanks (IkPas) in the Netherlands; these are referred to as ‘temporary alcohol abstinence challenges’ (TACs) (Alcohol Change 2021; IkPas. 2022; Minérale 2022). One of the aims of TACs is to encourage participants to break with their drinking behaviour (de Visser and Nicholls 2020). Officially participating in these challenges usually involves signing up voluntarily on the campaign website and receiving structured support and information.

The use of (external) support during TACs is considered to be essential. Participants who make (more) use of support have a better chance to complete the challenge successfully, change their drinking behaviour, and it may boost motivation (Pennay et al. 2018; de Visser and Nicholls 2020). Current support often consists of a personal dashboard on the campaign website, a forum to interact with other participants, and frequent newsletters (Bovens et al. 2021), yet still little is known about which support is most effective (for different participants) (de Visser and Nicholls 2020). As extra support, a self-help guide was developed by NoThanks that attempted to provide additional support to participants with a variety of information and self-help exercises in order to guide them through the TAC and ensure more long-term behavioural change.

This self-help guide is based on the transtheoretical model (TTM) of health behaviour change of Prochaska and colleagues (Prochaska et al. 1997). The model posits change as a dynamic process through several stages, although frequently not in a linear manner. The stages are characterized by dimensions of ‘readiness’ and proceed from not (yet) acknowledging that there is (problematic) behaviour that needs to be changed (precontemplation), to becoming aware of the problem (contemplation), to intending to take action soon (preparation), to making adjustments to lifestyle and environment (action), which leads to maintaining the change (maintenance) and no more temptation to turn back (*termination*). Further explanation of the self-help guide and the link with the theory can be found in the methods section.

The TTM was originally developed to explain how people change their addictive behaviours, such as smoking or drug use, and has been widely used to evaluate individual motivation for behavioural change (Senn et al. 2020). Critics of the model propose that stages are not mutually exclusive, behaviour is much more complex than just the six stages, and the model focuses largely on individual factors (Prochaska et al. 1997; Brug et al. 2005; Senn et al. 2020). On the other hand, the model was proven to be important in designing interventions to help people make behaviour changes due to its applicability and facilitating practical strategies (Velasquez et al. 2005).

Self-help interventions have been emerging as a promising approach to addressing alcohol-related problems (Andrade et al. 2016). The advantages of (online) self-help interventions include their low implementation costs, easy accessibility and availability (Sundström et al. 2017), and the possibility of providing guidance, advice, and strategies to participants with a view to changing their alcohol behaviour. They have proved to be effective in the general adult population (Riper et al. 2008). To the best of our knowledge, these kinds of self-help materials aimed at behavioural change have not yet been offered to participants in TACs.

TACs have been linked to reductions in alcohol consumption and improvements in (long-term) physical health and psychological well-being, such as sleep quality, energy levels, and general mental well-being (de Visser and Piper 2020; Bovens et al. 2021; Butters et al. 2023). Next to reductions in alcohol consumption, a temporary break from (excessive) drinking behaviour has been linked to increased drinking refusal self-efficacy (DRSE), which is the belief that one is able to refuse alcohol in different contexts (Young et al. 1991; de Visser and Nicholls 2020). This can happen through the experience of successful control of drinking behaviour, which potentially leads to long-term changes in this behaviour (Jenzer et al. 2021; Wood et al. 2023). Even a failed attempt to complete an abstinence challenge is associated with positive changes, such as increased self-efficacy, but smaller changes than participants who did complete the challenge (de Visser and Nicholls 2020).

Other elements that TACs could be linked to include behavioural automaticity (i.e. drinking habits) and craving (i.e. the urge to drink), given their associations with alcohol consumption (Albery et al. 2015; van Lier et al. 2018). Repeated alcohol consumption has been linked to increases in automatic behavioural responses and alcohol craving. Conversely, behavioural automaticity and craving are predictors of alcohol consumption (Gardner et al. 2012; Fleming and Bartholow 2014). Research has shown that participating in a TAC leads to fewer automatic behavioural responses and less alcohol craving (Pados et al. 2020).

The expectation is that participants with the extra support of the self-help guide during the TAC have a greater chance of successfully completing the TAC. Additionally, it is expected that they will report higher self-efficacy and reduced levels of behavioural automaticity, alcohol craving, and alcohol consumption after the TAC. The aim of this study is to investigate what the (added) effect of the self-help guide to the TAC is on (i) successful completion of the TAC and (ii) changes in DRSE, behavioural automaticity, craving, and alcohol consumption after 8 months.

## Methods

### Research design

A randomized controlled trial (RCT) design was used to examine the effectiveness of the self-help guide on drinking behaviour [trial registration number OSF.IO/B95VU (Kools and Bovens 2021)]. Quantitative data from a subgroup of NoThanks 2022 were used, which consisted of a baseline measurement and a follow-up after 8 months. The experimental group received the self-help guide; the control group did not. Participants were asked about their current alcohol consumption, drinking behaviour, self-efficacy, cravings, and various background characteristics (e.g. sex and age). This study was granted ethical approval by the Ethics Review Board of Tilburg University (TSB_RP229) and followed the CONSORT reporting guidelines (Schulz et al. 2010).

### Participants

Participants in NoThanks voluntarily signed up in December 2021 for the TAC and received online questionnaires before the abstinence period in January (T0) and 8 months after the abstinence period in September 2022 (T1).

**Figure 1:**
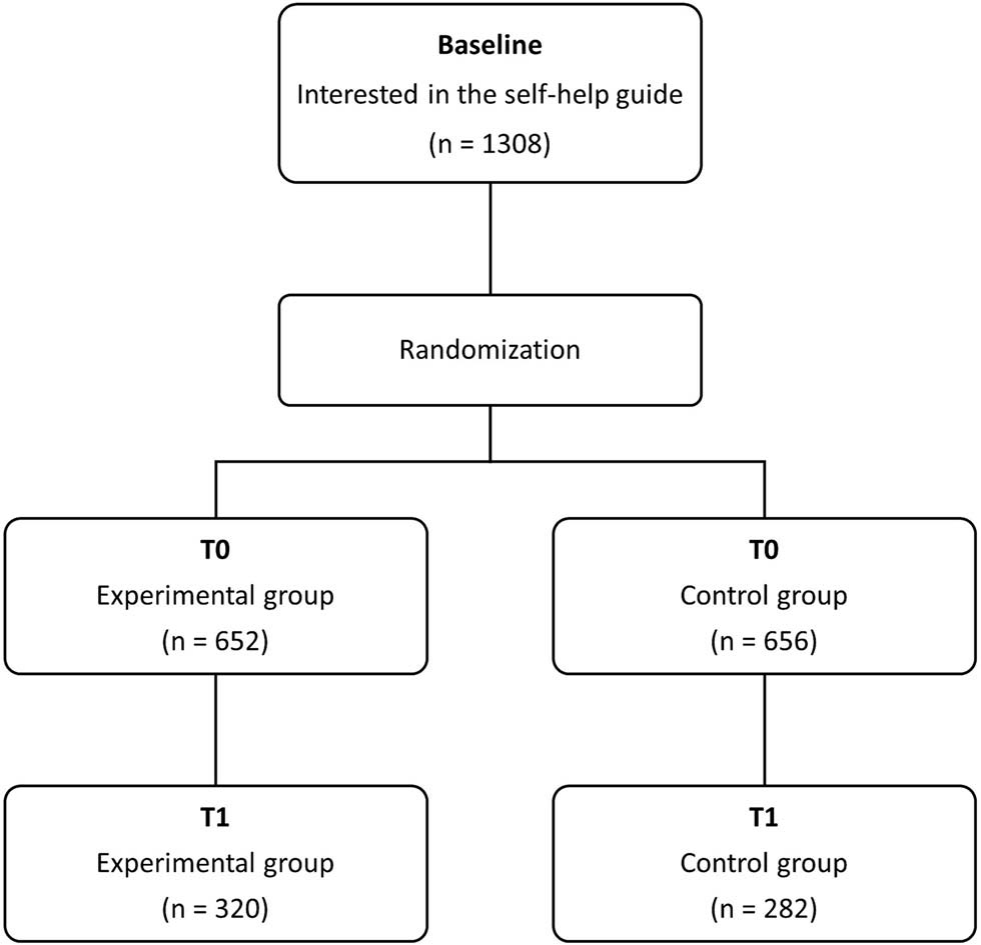
Flow diagram of the allocation of participants.

At the end of the first questionnaire, the first 4325 participants were asked whether they were interested in a self-help guide. Next, out of the 1308 participants (3.2%) who expressed their interest in the guide, 652 and 656 participants, respectively, were randomly assigned (stratified by sex) to either the experimental condition (i.e. NoThanks with self-help guide) or the control condition (i.e. NoThanks as usual). The follow-up questionnaire was completed by 602 participants (46%), of whom 320 belonged to the experimental group and 282 to the control group. Recruitment and allocation are shown in Fig. 1.

### Intervention

Participants allocated to the control group participated in the TAC as usual. They could make use of the standard support that was provided by the NoThanks organization. The participants assigned to the experimental group received the self-help guide via email. Since participants had already shown their intention to change when signing up for the TAC, the main aim was to provide information, skills, and the motivation to adopt other healthy behaviours and sustain this over time. The overall focus was on elements aimed at boosting self-efficacy and the belief in one’s ability to achieve one’s goals, as this has been found to be effective in stimulating positive changes in behaviour (Velasquez et al. 2015; Fergie et al. 2018).

At the start of every new week, beginning on 1 January 2022, participants received an email with a new chapter. The guide was structured into five chapters (see Table 1). Each chapter consisted of two to five pages explaining the theory behind a TTM stage, related to the context of changing drinking habits. Chapters contained exercises using behaviour-change techniques appropriate for the steps needed to be taken in the stage. These behaviour change techniques (the so-called ‘processes of change’) were designed to help participants to move from stage to stage, teaching them how to modify their thoughts, feelings, and behaviour (Prochaska et al. 1997; Marshall and Biddle 2001). It was emphasized to participants that everyone works at their own pace, and they could take more time for each chapter when needed. Completing exercises was optional, giving participants the freedom to only do the exercises they expected to be useful for themselves.

**Table 1:** Overview of the self-help guide.

**Chapter**	**TTM stage**	**Behaviour change techniques**	**Exercises**
1 Increasing the will to change	Contemplation	Consciousness raising Self-re-evaluation	Spider grams about mental/physical health Visualizing triggers
2 Reducing resistance to change	Contemplation	Self-re-evaluation	Disconfirm reasons to drink Visualizing triggers
3 Making plan to overcome obstacles	Preparation	Self-re-evaluation	Identifying old habits Create desired pattern
4 Going to change	Action	Self-liberation	Tracking progress Practising optimism
5 Sustaining change	Maintenance	Counterconditioning Reinforcement management Stimulus control	Guidance in exposure to challenging situations Coping with cravings

### Measures

The successful completion of the abstinence challenge was measured by asking the participants, ‘How many days did you drink alcohol during January?’ The answer options were: ‘I did not drink at all (0 days), 1 day, 2–3 days, or 4+ days’. These categories were combined with a binary distinction. This distinction consisted of (0) a successful completion and (1) no successful completion, i.e. the people who did drink one or more days.

DRSE was measured with the shorter version of the DRSE classification of the Tournée Minérale evaluation study (Thienpondt et al. 2017). This classification consisted of 11 items (instead of the original 19-item scale) and focused on three domains: social settings when other people are drinking (3 items), drinking for emotional regulation (4 items), and opportunistic drinking (4 items) (see Table 1 in Appendix B for the means of the separate domains). The participants were asked to rate their ability to resist drinking on a five-point Likert scale ranging from (1) ‘I am very sure I could not resist drinking’ to (5) ‘I am very sure I could resist drinking’. For the analysis, a total mean score of the 11 items was used (baseline Cronbach’s alpha = 0.92), with higher scores indicating a better DRSE.

Behavioural automaticity was measured by the Self-Reported Behavioural Automaticity Index (SRBAI) (Gardner et al. 2012). This index consisted of four items with statements, such as ‘alcohol consumption is something I do automatically’ or ‘without thinking’. The items were rated on a five-point Likert scale ranging from (1) ‘strongly disagree’ to (5) ‘strongly agree’. The total mean score of the four items was used (baseline Cronbach’s alpha = 0.75), with higher scores indicating more automatic behavioural responses.

Craving was measured with two questions, based on the first item of the Mini Alcohol Craving Experience Questionnaire (MACE) (Coates et al. 2017). This item was presented twice, where participants had to rate their craving to drink alcohol on weekdays and on weekend days. The items were rated on a five-point Likert scale ranging from (1) ‘I have no desire to use alcohol’ to (5) ‘I have an overwhelming desire to use alcohol’. The total mean score was based on these two items (baseline Cronbach’s alpha = 0.83).

Drinking frequency was measured by asking about the number of weekdays (Monday–Thursday) and weekend days (Friday–Sunday) on which alcohol was used. The answer categories consisted of ‘0 to <1 day’, ‘1 day’, ‘2 days’, ‘3 days’ (weekend days), and up to ‘4 days’ (weekdays). Drinking volume was measured by the usual consumed units on a weekday and on a weekend day. These categories consisted of 0–6 glasses as separate categories, ‘7–9 glasses’, and 10 glasses or more. The two categories that included a range of glasses, namely ‘7–9 glasses’ and ‘>10 glasses’, were recoded into the minimum number of glasses (respectively 7 and 10). Weekly alcohol consumption was calculated by adding the multiplication of the number of (week- and weekend) days on which alcohol was used to the number of usual consumed units on that week- and weekend-day. Finally, weekly alcohol consumption was reduced to two categories: (0) ‘non-excessive’ and (1) ‘excessive’*,* where excessive alcohol drinking consisted of more than 14 glasses (women) or 21 glasses (men) per week (Gezondheidsenquete/Leefstijlmonitor 2017)*.*

Different covariates were included. Sex, age, and educational level were also included in the analysis. Educational level was categorized into ‘low’ (i.e. primary school, intermediate secondary), ‘secondary’ (i.e. higher secondary/preparatory university education), and ‘high’ (i.e. higher professional education, university). Additionally, whether participants who did receive the self-help guide used it during the TAC was measured by the question: ‘Have you taken the opportunity to use the self-help guide with the variety of exercises to break habitual behaviours?’. Answer categories consisted of ‘No’, ‘Yes, during the abstinence period’, and ‘Yes, even after January’*.* To make a binary distinction, the last two categories were labelled ‘*Yes*’.

### Statistical analysis

The data were analysed using IBM SPSS Statistics 27. To examine changes between baseline and follow-up, only the participants who participated in both measurements were analysed (*n* = 602). Before the analysis, unpaired *t*-tests and chi-square tests were conducted to test for possible differences between the experimental and control groups in individual characteristics and in scores at baseline on DRSE, behavioural automaticity, craving, and alcohol consumption.

First, logistic regression analyses were used to measure the difference in likelihood of a successful completion of the TAC between the experimental group and the control group. Second, both linear mixed models (LMMs) and generalized linear mixed models (GLMMs) were used to measure the change in scores on DRSE, behavioural automaticity, and craving, and in the probability of being an excessive drinker between baseline and follow-up. The main statistical analysis was performed according to the intention-to-treat (ITT) principle, that is, according to the treatment allocation at randomization. The control group (0) was used as the reference group, which was compared with the experimental group (1). The univariable models consisted of the intervention and time of measurement as fixed factors, including the interaction between intervention and time of measurement, to test whether the change over time differed between the experimental and control groups. In the multivariable models, the different covariates were included (age, sex, educational level, and successful completion of the TAC). Sensitivity analyses were performed in which the experimental group was split into the group that used the guide and the group that did not, and both were compared with the control group.

## Results

Unpaired *t*-tests and chi-square tests showed several statistically significant differences between dropouts (*n* = 706) and completers of both measurements (*n* = 602). Differences were found for age (*t* = −11.06, *P* < .001), education (χ^2^ = 12.34, *P* < .01), weekly alcohol consumption (χ^2^ = 9.15, *P* < .01), DRSE (*t* = −2.55, *P* < .05), and behavioural automaticity (*t* = 2.91, *P* < .01). Dropouts from the study were more likely to be younger, less educated, excessive drinkers, and to have a lower DRSE and higher behavioural automaticity than completers.

Baseline and follow-up characteristics are presented in Table 2. Most participants were female in both groups (around 57%). The mean age at baseline was 3–4 years lower than at the follow-up. Around 70% were highly educated. In both groups, ~54% were identified as excessive drinkers (>14 or 21 glasses per week, respectively, for women and men). Of the participants allocated to the experimental group, 42.5% used the self-help guide, and 9.6% kept using the guide after the TAC. Unpaired *t*-tests and chi-square tests showed that the experimental and control groups were not statistically different sex, age, educational level, and baseline measurements.

**Table 2:** Characteristics of the experimental and control group at baseline and follow-up.

	**Baseline**		**Follow-up**			
	Experiment	Control	Experiment	Control		
	*n* = 1308		*n* = 602		Min.	Max.
Sex, no (%)						
Male	282 (43.3)	278 (42.5)	142 (44.3)	122 (43.3)		
Female	370 (56.7)	376 (57.3)	178 (55.6)	159 (56.4)		
Age, mean (SD)	53.80 (11.88)	53.70 (12.18)	57.12 (1.04)	57.93 (11.03)	18	84
Education, no (%)						
Low	43 (6.6)	44 (6.7)	19 (5.9)	16 (5.7)		
Secondary	152 (23.3)	149 (22.7)	63 (22.4)	53 (18.9)		
High	457 (70.1)	462 (70.4)	238 (74.4)	212 (75.4)		
Successful completed, no (%)						
Yes			222 (69.4)	182 (64.8)		
No			98 (3.6)	99 (35.2)		
Drinking refusal self-efficacy, mean (SD)	3.95 (.73)	3.96 (.72)	4.13 (.79)	4.03 (.82)	1	5
Behavioural automaticity, mean (SD)	3.18 (.82)	3.13 (.80)	2.27 (.83)	2.38 (.87)	1	5
Craving, mean (SD)	2.55 (.81)	2.60 (.80)	2.23 (.73)	2.26 (.76)	1	5
Weekly alcohol consumption, no (%)						
Non-excessive	302 (46.3)	300 (45.7)	220 (68.8)	186 (66.0)		
Excessive^a^	350 (53.7)	356 (54.3)	100 (31.3)	96 (34.0)		
Use self-help guide, no (%)						
Yes			136 (42,5)			
No			184 (57,5)			

^a^Excessive alcohol drinking is 14 (women) or 21 (men) or more glasses per week.

The logistic regression showed no statistically significant difference in the likelihood of successfully completing the TAC between the experimental group and the control group. Participants of the experimental group did not have a greater chance of completing the TAC successfully than participants of the control group (OR = 0.83, CI = 0.59–1.16).

The LMM analyses for DRSE, behavioural automaticity, and craving are shown in Table 3. At the follow-up, the participants of both groups scored significantly lower in behavioural automaticity and craving than at baseline. In all multivariable models, no significant differences were found between the experimental group and control group. Additionally, the interactions between the group and time of measurement were not significant; the change over time was not statistically significantly different between the two groups.

**Table 3:** Overall effectiveness self-help guide on DRSE, behavioural automaticity and craving.

	**DRSE**		**Behavioural automaticity**		**Craving**	
**Variables**	**Univariable model** **Estimate (95% CI)**	**Multivariable model** **Estimate (95% CI)**	**Univariable model** **Estimate (95% CI)**	**Multivariable model** **Estimate (95% CI)**	**Univariable model** **Estimate (95% CI)**	**Multivariable model** **Estimate (95% CI)**
Time of measurement	−.07 (−.02–.16)	0.08 (−.01–.17)	−.70^*^^*^ (−.81—.59)	−.68^*^^*^ (−.79—.57)	−.39^*^^*^ (−.48—.31)	−.38^*^^*^ (−.47—.30)
Group (ref. = ctr)	−.09 (−.03–.21)	0.07 (−.05–.19)	0.04 (−.10–.17)	0.04 (−.09–.17)	−.13^*^ (−.26—.01)	−0.12 (−.25–.00)
Group^*^time	−.00 (−.12–.12)	−.01 (−.13–.11)	−.13 (−.27–.02)	−0.14 (−.29–.01)	0.12 (.00–.23)	0.10 (−.02–.22)
Age		−.00 (−.01–.00)		−.01^*^^*^ (−.01—.00)		-.00 (−.01–.00)
Sex (ref. = male)		−.08 (−.19–.03)		−0.19^*^^*^ (−.31—.08)		0.02 (−.09–.13)
Education (ref. = low)						
* Secondary*		−0.16 (−0.41–.09)		0.20 (−.08–.47)		−.11 (−.38–.15)
* High*		−.04 (−.27–.19)		0.23 (−.02–.48)		−0.28^*^ (−.52—.4)
Completion (ref. = yes)		−0.29^*^^*^ (−.40—.18)		0.26^*^^*^ (.14–.38)		0.23^*^^*^ (.11–.35)

^*^ *P* < .05, ^*^^*^p < .01.

As shown in Table 4, GLMM analysis showed that the probability of being an excessive drinker was significantly lower at follow-up than at baseline. This indicated that the percentage of excessive drinkers decreased over a period of 8 months. There were no significant differences between the experimental and control groups; the probability of being an excessive drinker was not significantly different between the experimental and control groups. Moreover, the interaction between the group and time of measurement was not significantly different, meaning that the degree of change over time was not statistically different between the two groups.

**Table 4:** Overall effectiveness self-help guide on non-excessive/excessive alcohol consumption.

**Variables**	**Univariable model** **ORs (95% CI)**	**Multivariable model ORs (95% CI)**
Time of measurement	0.41^*^^*^ (.29–.60)	0.41^*^^*^ (.28–.60)
Group (ctr)	0.83 (.55–1.26)	0.84 (.55–1.28)
Group^*^time	1.04 (.62–1.74)	1.04 (.62–1.76)
Age		1.01 (1.00–1.03)
Sex (male)		0.51^*^^*^ (.36–.72)
Education (ref. = low)		
*Secondary*		0.95 (.42–2.14)
*High*		0.70 (.34–1.47)
Completion (yes)		0.78 (.54–1.13)

^*^ *P* < .05,^*^^*^P < .01.

Two sensitivity analyses were performed. For these analyses, the experimental group was divided into two groups, consisting of those respondents who used the guide (*n* = 136) and those who did not (*n* = 184). The logistic regression (Table 2 in Appendix B) showed no statistically significant differences in the likelihood of successfully completing the TAC between the three groups.

Additionally, no statistically significant differences were found in changes over time in DRSE, or behavioural automaticity, or in the probability of being an excessive drinker between the participants of the three groups (Tables 3 and 4 in Appendix B). The participants allocated to the experimental group who did not use the self-help guide did show a significantly lower craving than the control group. The statistically significant interaction effect with time showed that the control group had a larger decrease in craving than the group that did not use the self-help guide.

## Discussion

### Key findings

The present study aimed to evaluate the additional effect of a self-help guide on a TAC and found no such significant additional effect. Participants who did receive the self-help guide did not differ in their probability of completing the TAC successfully or in the degree of change in DRSE, behavioural automaticity, craving, and alcohol consumption between baseline and the 8-month follow-up from the participants who did not receive the guide.

### Strengths and limitations

One strength of this study was that it evaluated a self-help intervention as extra support during another intervention, while previous studies on self-help alcohol interventions generally involved a comparison with either another behavioural intervention or no intervention (Sundström et al. 2017). Another strength was the randomized and controlled study design, which resulted in a comparable experimental and control group. Moreover, this study included a long-term follow-up, which is not always feasible in research.

However, some limitations need to be acknowledged. First, the implementation fidelity was low. Although the participants included in the study did indicate a need for more support, 42.5% ended up not using it. There was a lack of information about the exposure of the experimental group to the guide and why most of them stopped or did not even start using it during the TAC. Second, only 30% of the survey respondents on the baseline questionnaire were interested in the self-help guide. Therefore, the need for this form of support did not seem high. While there were several advantages of the guide (e.g. easy accessibility and low cost), the self-help guide was developed based on theory (Prochaska et al. 1997), and a needs assessment prior to developing the self-help guide would have been valuable to identify the needs of TAC participants in terms of extra support. For further research, the needs of participants who were interested in more in-depth support should be examined to develop a better customized and bottom-up implemented form of extra support for the TAC. The last limitation is that the study had a high dropout rate, though this is common for (web-based) self-help interventions (Andrade et al. 2016). This high attrition may have biased the overall results. Differences were found between the study dropouts and completers of both questionnaires. Since the dropouts were younger and excessive drinkers, they would probably have benefited most from the intervention, as similar interventions work better for them (White et al. 2010). It would be interesting to study in future research whether a self-help guide works for this group of participants.

### Interpretation of findings

Contrary to expectations, no effect of adding the self-help guide to the TAC was found. There may be some reasons for the intervention not being effective. It is important to note that no causal effects of TACs on alcohol consumption can be determined. First, participants in TACs are generally excessive drinkers who have a high motivation to change their drinking behaviour (demonstrated by their voluntary participation in a TAC), which might explain their alcohol behaviour change (de Visser and Nicholls 2020; Butters et al. 2023) rather than their participation in the TAC. Second, it was not possible to compare TAC-participants to a control group that was not exposed to the national campaign NoThanks.

When interpreting the results, it should be noted that they may differ from other studies, since the present study compared additional support to the standard intervention with regular support rather than comparing support to no support. Comparable unguided online self-help interventions mostly have a small, but significant, effect on alcohol consumption (Webb et al. 2010; Riper et al. 2014; Sundström et al. 2017; Riper et al. 2018). These online self-help interventions, however, generally work best for young people, women, people with a higher educational level, and excessive drinkers (Riper et al. 2008; White et al. 2010). They are less effective for older adults, which is noteworthy given that the study population in the sample of this study is relatively old.

Another explanation could be that the duration of the TAC (1 month) is too short for a self-help intervention using behaviour change techniques (Andrade et al. 2016). Although interventions that incorporate behaviour change techniques are more effective than other interventions (Webb et al. 2010), they are also more extensive and intensive, and it takes longer for participants to integrate. Similar interventions mostly take 6–12 weeks (Riper et al. 2014). Additionally, the self-help guide started in the contemplation stage. The assumption that all participants started in the same stage, including the fact that it was not customized to the participants position and needs, might also be a reason participants did not use the guide or stopped using it. Developing a version of the intervention designed for a longer period of time with more guidance during the behaviour change techniques, such as using interactive elements or automated tailored feedback, might enhance the effectiveness for TAC participants (Webb et al. 2010).

Since the study population consisted of the first people to register for NoThanks, they were likely to be the most motivated participants in the TAC. This might limit the generalizability of the average TAC participant. However, these participants were chosen because motivation was needed to put extra hours aside each week for reading and exercises. Their motivation to change their drinking behaviour was high, and therefore they might have used a lot of the other support from the organisation. However, there was no opportunity to control for this, since there was no information available about the other (standard) support used by the participants within both the experimental and control groups. It was thus unclear whether participants within the experimental group replaced the standard support or used the self-help guide as extra support. It would be interesting in future studies to control for the use of other support offered by the NoThanks organization.

A larger decrease in cravings was found in the control group than in the experimental group that did not use the guide. This might be because the measurement of craving was limited to only one item of the standardized MACE and thus might not be completely reliable. Additionally, due to conceptual confusion, measuring craving has shown inconsistent results in the literature, as it is not always predictive of (relapsing to) alcohol consumption (Kavanagh et al. 2013; Serre et al. 2015). Further research is needed to interpret this finding.

## Conclusion

Overall, the findings indicated no substantial additional effect of the self-help guide on the TAC. This is potentially due to the design of the self-help guide and/or the characteristics of the study population. It would be worthwhile examining the needs of participants in TACs and what they think they need to ensure long-term change in their alcohol consumption, and focusing on a bottom-up process in developing a self-help intervention.

**Table:** 

		Reporting Item	Page Number
Ancillary analyses	#18	Results of any other analyses performed, including subgroup analyses and adjusted analyses, distinguishing pre-specified from exploratory	12
Harms	#19	All important harms or unintended effects in each group (For specific guidance see CONSORT for harms)	n/a
**Discussion**			
Limitations	#20	Trial limitations, addressing sources of potential bias, imprecision, and, if relevant, multiplicity of analyses	13
Generalisability	#21	Generalisability (external validity, applicability) of the trial findings	14
Interpretation	#22	Interpretation consistent with results, balancing benefits and harms, and considering other relevant evidence	14
Registration	#23	Registration number and name of trial registry	5
**Other information**			
Interpretation	#22	Interpretation consistent with results, balancing benefits and harms, and considering other relevant evidence	14
Registration	#23	Registration number and name of trial registry	5
Protocol	#24	Where the full trial protocol can be accessed, if available	16
Funding	#25	Sources of funding and other support (such as supply of drugs), role of funders	

## Data Availability

The data underlying this article are available on reasonable request to the corresponding author.

## Appendix A. CONSORT guidelines

**Table:**

		Reporting Item	Page Number
**Title and abstract**			
Title	#1a	Identification as a randomized trial in the title	1
Abstract	#1b	Structured summary of trial design, methods, results, and conclusions	2
**Introduction**			
Background and objectives	#2a	Scientific background and explanation of rationale	3
Background and objectives	#2b	Specific objectives or hypothesis	4
**Methods**			
Trial design	#3a	Description of trial design (such as parallel, factorial) including allocation ratio	5
Trial design	#3b	Important changes to methods after trial commencement (such as eligibility criteria), with reasons	n/a
Participants	#4a	Eligibility criteria for participants	5
Participants	#4b	Settings and locations where the data were collected	5
Interventions	#5	The experimental and control interventions for each group with sufficient details to allow replication, including how and when they were actually administered	6
Outcomes	#6a	Completely defined prespecified primary and secondary outcome measures, including how and when they were assessed	7
Outcomes	#6b	Any changes to trial outcomes after the trial commenced, with reasons	n/a
Sample size	#7a	How sample size was determined	5
Sample size	#7b	When applicable, explanation of any interim analyses and stopping guidelines	n/a
Randomization - Sequence generation	#8a	Method used to generate the random allocation sequence	5
Randomization - Sequence generation	#8b	Type of randomization; details of any restriction (such as blocking and block size)	5
Randomization - Allocation concealment mechanism	#9	Mechanism used to implement the random allocation sequence (such as sequentially numbered containers), describing any steps taken to conceal the sequence until interventions were assigned	5
Randomization - Implementation	#10	Who generated the allocation sequence, who enrolled participants, and who assigned participants to interventions	5
Blinding	#11a	If done, who was blinded after assignment to interventions (for example, participants, care providers, those assessing outcomes) and how	n/a
Blinding	#11b	If relevant, description of the similarity of interventions	n/a
Statistical methods	#12a	Statistical methods used to compare groups for primary and secondary outcomes	8
Statistical methods	#12b	Methods for additional analyses, such as subgroup analyses and adjusted analyses	8–9
**Results**			
Participant flow diagram (strongly recommended)	#13a	For each group, the numbers of participants who were randomly assigned, received intended treatment, and were analysed for the primary outcome	6
Participant flow	#13b	For each group, losses and exclusions after randomization, together with reason	6
Recruitment	#14a	Dates defining the periods of recruitment and follow-up	5
Recruitment	#14b	Why the trial ended or was stopped	5
Baseline data	#15	A table showing baseline demographic and clinical characteristics for each group	10
Numbers analysed	#16	For each group, number of participants (denominator) included in each analysis and whether the analysis was by original assigned groups	6–8
Outcomes and estimation	#17a	For each primary and secondary outcome, results for each group, and the estimated effect size and its precision (such as 95% confidence interval)	11
Outcomes and estimation	#17b	For binary outcomes, presentation of both absolute and relative effect sizes is recommended	11

## Appendix B. Sensitivity analyses

**Table B1:** Mean (SD) of each subscale of DRSE.

	**Baseline**		**Follow-up**	
	Experiment	Control	Experiment	Control
Social settings	3.92 (.88)	3.95 (.89)	4.11 (.93)	3.97 (.96)
Emotional regulation	3.62 (1.02)	3.62 (1.02)	2.85 (1.11)	3.75 (1.15)
Opportunistic drinking	4.30 (.67)	4.31 (.66)	4.41 (.68)	4.35 (.74)

*^*^* No statistically differences were found in changes in scores on the three separate domains of DRSE between the experimental and control group.

**Table B2:** Effectiveness self-help guide on (successful) completion of TAC for the different subgroups.

**Variables**	**Univariable model**	**Multivariable model**
	**ORs (95% CI)**	**ORs (95% CI)**
Constant	0.54^*^	0.33^*^
Group (ref. = ctr)		
Exp^a^	0.87 (.59–1.29)	0.88 (.60–1.31)
Exp^b^	0.74 (.47–1.15)	0.75 (.48–1.18)
Age		1.00 (.98–1.02)
Sex (ref. = male)		1.53^*^ (1.07–2.17)
Education (ref. = low)		
Secondary		0.95 (.76–1.18)
High		1.06 (.85–1.32)

^a^Respondents from experimental group who didn’t use the guide.

^b^Respondents from experimental group who did use the guide.

^*^ *P* < .05. ^*^^*^*P* < .01.
